# One trait, many signals: different information on male quality is enclosed within the same trait in a blenny fish

**DOI:** 10.1007/s00114-012-0959-4

**Published:** 2012-08-17

**Authors:** Lisa Locatello, Matteo Pizzolon, Maria Berica Rasotto

**Affiliations:** Department of Biology, University of Padova, via U. Bassi 58/B, Padova, Italy

**Keywords:** Carotenoids, Multicomponent traits, Condition, Fish, Sexual selection

## Abstract

Colourful ornaments are traditionally evaluated as one trait. However, they could consist of several components, such as total size, colour intensity and extension, each possibly bearing its own message about one aspect of quality. Males of the blenny *Salaria pavo* exhibit a colourful head crest and solely care for eggs. During the breeding season, the head crest shows a yellow colouration, the intensity and relative extension of which are independent of crest size. Here, we show that: (1) carotenoids are responsible for the head crest yellow patch; (2) activating the immune system by injecting the bacterial antigen lipopolysaccharides affects both the intensity and extent of the yellow colouration; and (3) females assess males on the basis of colour patch expression. However, the response of the yellow patch to the immune challenge was dependent on head crest size. Indeed, males with a larger head crest reacted better to the simulated infection, sustaining a level of yellow patch close to pre-challenge size.

**Electronic supplementary material:**

The online version of this article (doi:10.1007/s00114-012-0959-4) contains supplementary material, which is available to authorized users.

## Introduction

In many species, females base their mate choice on the simultaneous recognition of multiple signals, rather than on one character only. Why this occurs is still debated, and several contrasting hypotheses were proposed to explain how female preference for multiple traits can arise and persist (Candolin 2003; Hebets and Papaj 2005; Bro-Jorgensen 2010). Experimental data are necessary to progress in the understanding of female choice, and a key step consists in the careful assessment of the information conveyed by male traits. In this respect, particular attention is given to those traits, such as colourful ornaments, that are traditionally regarded as a single signal but may consist of several components that can encompass different types of information regarding the signaller (Candolin 2003; Grether et al. 2004). Indeed, ornament size could reflect condition over a longer time scale or genetic quality, depending on past growth or genetic control, whereas colour expression might be more flexible and indicate present condition (Candolin 2003; Grether et al. 2004).

In the peacock blenny, *Salaria pavo*, males exhibit multiple sexual traits and perform parental care (Oliveira et al. 1999). The mating system is promiscuous: males receive eggs from different females, simultaneously caring for overlapping clutches, and females spawn with several males during the breeding season. Nesting males are larger than females and exhibit two sexually dimorphic traits (Fig. S1 in Online resource 2), whose expression is positively related to body size: a pronounced head crest and the first two rays of the anal fin transformed into a pair of glands (Oliveira et al. 1999). Experimental evidences show that females intensively court males with more developed head crests (Gonçalves et al. 2002) and care for anal gland size when crest size is controlled (Pizzolon et al. 2010). Head crest is a multicomponent trait as, during the breeding season, it shows a yellow patch on both sides, the brightness and extension of which are unrelated to the crest size. In many animal species, yellow ornamentations are carotenoid based and are viewed as an honest signal of the bearer’s present condition (Vinkler and Albrecht 2010; Svensson and Wong 2011). If the head crest yellow patch of *S. pavo* males is also carotenoid based and conveys information on the phenotypic quality of the male at the time of mating, we expect changes in the individuals’ health to be reflected by changes in patch expression. Considering that males in poor condition eat the eggs in their nest in order to improve their physical condition (Barata et al. 2008; Pizzolon et al. 2010), we also expect females to prefer more colourful partners, as they appear healthier. Moreover, as in species where ornaments reflect genetic and/or long-term phenotypic quality, more ornamented males better cope with parasite load (Wedekind 1992); if head crest size reflects long-term condition, we expect males with a larger crest to better face the effects of infection. To test these expectations, we:

1. Confirmed the presence of carotenoids in head crest yellow patches.
2. Report on variation in male displays in response to an immune system challenge, through the injection of lipopolysaccharides (LPS), an *Escherichia coli* antigen that rapidly induces oxidative stress (Bonneaud et al. 2003). To control that the experimental manipulation affects only head crest characteristics, we evaluated also anal gland size and male behaviour.
3. Assess female preferences for yellow crest patch expression.

## Materials and methods

Fifty-eight males (total length, TL, 8.30–14.38 cm) and 42 females (TL, 5.34–11.61 cm) were captured in the Venetian Lagoon during the breeding season (July–August) 2011 (details on capture and maintenance in Online resource 1).

### Head crest pigment characterization

Two males were killed with an overdose of MS222 (tricaine sulphate, Sandoz), and extracts of their head crest tissue were subjected to reverse-phase high-performance liquid chromatography (HPLC) analyses (details in Online resource 1).

### Immune-challenge experiment

The day after capture (day 1), anal gland area, head crest size (height, area and thickness) and yellow patch area and colour intensity of each male were measured (detailed methods in Online resource 1). A blood sample (20–100 μl) was collected to determine the baseline health status of all individuals with assay of plasma nitric oxide (NO) concentration (Oxford Biomedical Research kit, Online resource 1 for details). Males were then left undisturbed for 2 days, and on day 4 and 5, they were presented, twice a day, for 10 min each time, to a ready-to-spawn female to register courtship behaviours (time spent outside the nest over total observation time, number of attacks on females and reaction time to females’ presence). On day 6, males were injected and randomly assigned to treated (*n* = 21) or control groups (*n* = 21). The two male groups did not differ in the baseline values of the recorded traits (morphological traits, behaviours and health status) (*t* test: all *p* > 0.17). The immune treatment consisted of an intramuscular injection of 4 ml/100 g body weight of LPS (serotype: O26:B6; Sigma-Aldrich) diluted in 0.005 % phosphate-buffered saline (PBS), corresponding to a dose of 2 mg/kg. Control males were injected with the same volume of PBS. A preliminary test on seven controls and seven treated males, through the measurement of plasma NO concentration, before LPS/PBS injection and 24 hours later, demonstrated the rapid effect of LPS dosage in our target species (detailed results in Online resource 1). Males were left undisturbed on day 7 and 8. On day 9 and 10, we collected the post-treatment observations on courtship behaviours, and on day 11, the post-treatment measurement of male morphological traits was performed as described above.

### Mate-choice experiment

Briefly, the experiment was performed in partitioned tanks using male dummies differing only for the yellow patch extension and/or intensity (detailed experimental procedure in Online resource 1; Fig. S1 and S2 in Online resource 2). Three sets of trials were performed to test female preference for two dummies differing in: (1) yellow patch area (*n* = 15), (2) yellow patch intensity (*n* = 13) and (3) both patch area and intensity (*n* = 14), with one dummy exhibiting a patch with larger area and lower colour intensity, whilst the patch of the other had opposite features.

### Statistical analyses

Data were checked for normality using a Kolmogorov–Smirnov test and log-transformed when appropriate. The effect of immune activation on male traits was assessed using an ANOVA for repeated measures. Female mate-choice preference was calculated as the proportion of time spent in front of a given dummy relative to the total time spent in the choice zones (Pizzolon et al. 2010). Proportions were arcsine transformed and tested against an expectation of no preference (proportion of time = 0.5) with a one-sample test.

## Results

### Head crest pigment characterization

HPLC revealed a peak of retention time at 16 min and three peaks of absorbance at 416, 440 and 468 nm. According to reference spectra (Jeffrey et al. 1997), these findings confirm that the yellow colouration is carotenoid based and primarily due to ε,ε carotene.

### Immune-challenge experiment

**Fig. 1:**
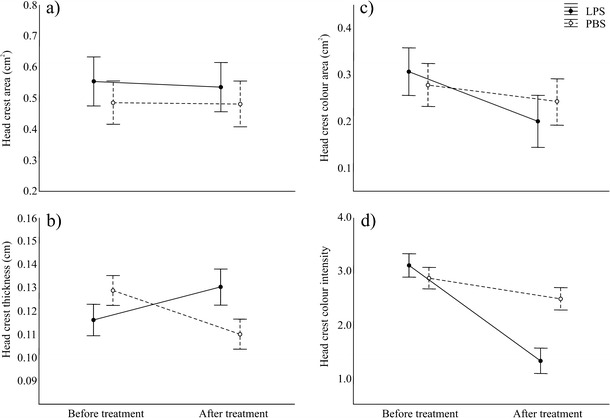
Effect of the injection of LPS (immune-treated males) or PBS (control males) on **a** head crest area, **b** head crest thickness, **c** head crest colour area, and **c** head crest colour intensity. Data are shown as mean ± SE

Most of the analysed traits showed a significant variation in time regardless of treatment (Table 1) (mean data in Online resource 1), suggesting a general effect of manipulation. Nevertheless, we observed a higher reduction of head crest characteristics (height, total area, yellow patch area and intensity) in LPS-injected males than in controls (Table 1; Fig. 1). By contrast, crest thickness increased in LPS-treated males and decreased in controls (Table 1; Fig. 1). The proportional change in head crest area, height and thickness (calculated as value after treatment minus value before treatment divided by value before treatment) was not related to male body size (TL) (all *r* < 0.28; all *p* > 0.07).

**Table 1:** Results of repeated measure ANOVA on male traits before and after (“time”) the injection of LPS in treated males or PBS in control males (“treatment”)

	Treatment		Time		Time × treatment		
	F	P	F	P	F	P	d.f.
Morphological traits							
Body weight	0.96	0.33	25.54	<0.001	0.01	0.94	1.40
Head crest area	0.25	0.62	11.91	0.013	4.53	0.040	1.40
Head crest thickness	0.22	0.64	0.47	0.50	21.28	<0.001	1.40
Head crest height	0.02	0.88	14.96	<0.001	4.67	0.049	1.40
Head crest colour area	0.13	0.73	22.95	<0.001	6.52	0.035	1.40
Head crest colour intensity	4.25	0.049	55.40	<0.001	21.66	<0.001	1.40
Anal gland area	1.24	0.27	59.10	<0.001	1.70	0.20	1.40
Behavioural traits							
Time outside the nest	0.10	0.75	0.53	0.47	0.95	0.34	1.40
No. of attacks to female	0.01	0.91	11.77	0.002	2.16	0.15	1.40
Reaction time to female	0.04	0.83	14.74	<0.001	1.97	0.17	1.40

Directions of the time × treatment effects in Fig. 1

To test the expectation that, if head crest size reflects male long-term condition, males with larger head crests should better respond to the simulated infection, we correlated the proportional change in head crest characteristics with head crest area. We found that crest total area was positively correlated to the proportional change in yellow patch area (*r* = 0.56; *p* = 0.009), with males exhibiting smaller head crests suffering the strongest LPS-induced reduction in patch area, whilst it was unrelated to changes in colour intensity, crest height and thickness (all >0.18).

### Mate-choice experiments

Females showed a clear preference for dummy with larger (trial 1) and more intense (trial 2) yellow patches, spending respectively an average of 75.3 ± 24.7 and 85.6 ± 14.4 % SE of the total choice time in front of them (one-sample *t* test: patch area *t*_10_ = 6.22; *p* < 0.001; patch intensity *t*_10_ = 14.05; *p* < 0.001). In the third trial, females spent an average of 75.4 ± 14.4 % SE of total choice time in front of dummy with larger patch area and lower colour intensity (one-sample *t* test: *t*_9_ = 2.65; *p* = 0.026).

## Discussion

Our results show that the crest yellow patch of *S. pavo* males can be used by females to predict male current health status. Indeed, males’ response to an immune challenge is signalled by a decrease in the extension and intensity of crest yellow colouration, whereas other displays, such as anal glands and behaviours, remain unaffected by the treatment. These findings suggest that in this blenny, as it occurs in other species (Vinkler and Albrecht 2010; Svensson and Wong 2011), carotenoids might be mobilized in response to the oxidative stress caused by infection. Head crest also slightly swelled in response to immune activation, causing an increase in thickness and a consequent reduction in height. This is due to the oedema, associated to the inflammatory response evoked by LPS (Passos et al. 2004). Hence, a reduction in crest area does not necessarily occur every time the health of an individual deteriorates.

Males with a larger head crest seem to better respond to the simulated infection, as they maintain the size, but not the colour intensity, of the yellow patch closer to that exhibited before the challenge. This finding suggests that head crest, like other multicomponent traits (Candolin 2003), may convey information on male condition over different time scales, with its general development reflecting genetic and/or condition at the time of formation and growth, while colour intensity signals current health status.

Peacock blenny females assess prospecting mates on the basis of all morphological traits, but they attribute greater weight to head crest size (Gonçalves et al. 2002). Our tests show that females are able to discriminate against males also on the basis of head crest yellow patch expression, a trait revealing differences between males in condition. In *Poecilia reticulata*, a fish species with a non-resource-based mating system, female choice for colourful males seems to be driven by both direct benefits, such as the reduced probability of contracting parasites or disease and/or indirect (genetic) ones, as increased offspring performance and enhanced reproductive performance of male offspring (reviewed in Pilastro et al. 2008). However, in species where male condition is crucial for determining offspring survival, such as in *Gasterosteus aculeatus*, females gain direct fitness benefits in choosing colourful partners given their greater likelihood of surviving the breeding attempt (Pike et al. 2007). Similarly, in the peacock blenny, females using colour expression as a proxy of male condition might maximize their chances of mating with a healthy male, who will rear offspring to independence, without cannibalizing them.

## Electronic supplementary material

Below is the link to the electronic supplementary material.

ESM 1 (PDF 112 kb)

ESM 2 (PDF 601 kb)

## Abbreviations

LPS: Lipopolysaccharides
PBS: Phosphate-buffered saline
NO: Nitric oxide
HPLC: High-performance liquid chromatography
