# Supplementary material for: One trait, many signals: different information on male quality is enclosed within the same trait in a blenny fish
Source: Naturwissenschaften. 2012 Aug 17;99(10):863–7. doi: 10.1007/s00114-012-0959-4 (PMC3448905; doi:10.1007/s00114-012-0959-4)
Supplement: Supplementary file 1 — (PDF 112 kb) [file 114_2012_959_MOESM1_ESM.pdf]

One trait, many signals: different information on male quality is enclosed within the same trait in a blenny fish

Lisa Locatello, Matteo Pizzolon, Maria Berica Rasotto

SUPPLEMENTARY MATERIAL AND METHODS

Fish capture and maintenance

Fish were capture by scuba-divers using hand-held nets, and immediately transported to the lab. Males were kept individually in 36L tanks and females in group of three/four individuals in 70L tanks. All tanks were provided with sandy bottoms and artificial shelters. Females were transferred into individual experimental tanks (Fig. S2 in Online Resource 2) for two days of acclimatization before trials. Water was renewed daily, temperature was maintained between 18 and 22°C, and the light regime followed natural conditions. Fish were fed daily with fresh chopped *Mytilus sp*.

Head-crest pigment characterization

Extracts of head-crest tissue were obtained through overnight incubation in 100% chilled acetone and were used for reversed phase HPLC (high-performance liquid chromatography) analyses, following methods detailed described in Färber and Jahns (1998). Solvents used for pigments extraction were composed of acetonitrile, methanol, and 0.1M Tris/NaOH pH 8 in a ratio of 87:10:3 (solvent A) and a 4:1 mixture of methanol and hexane (solvent B). Eluted pigments were monitored by their absorption at 440 nm.

Immune challenge experiment

Measure of male morphological traits

To assess the yellow patch colour intensity each male was housed for 10 minutes in a transparent glass box and compared to a Pantone colour scale fixed on the box wall. Each male was assigned a value ranging from 0, absence of colour, to 4, maximum colour intensity (1= R 255, G 200, B 000; 2= R 255, G 210 B 047; 3= R 225 G 220 B 91, 4= R 225, G 230, B 139). This comparison was performed on free swimming males to avoid problems of colour fade that typically occurs after anaesthesia. Each male was then anesthetized in a water solution of MS222 (Tricaine sulphate,

Sandoz) and weighted to the nearest 0.01 g. A digital photo was taken of the male alongside a reference ruler on both his lateral and frontal side and, using image analyses on digital photos, we measured ì) male body size (TL) ìì) anal-gland area, ììì) head-crest height, area and thickness, ìv) yellow-patch area.

Nitric oxide assay and effect of LPS dosage

Nitric oxide is a multifunctional molecule that increases during inflammatory processes, and an estimation of its production provides a measure of individual variation in physiological condition, health status, and work load (Sild & Hõrak 2009).

In order to assess the health status of all individuals before the experimental process and to demonstrate the effect of LPS dosage (2 mg/Kg) we evaluated the nitric oxide concentration in plasma. In biological tissues NO has a half-life of few seconds and therefore its production is assessed on the basis of its stable oxidation end products, nitrate and nitrite. The NO assay was performed with a non-enzymatic colorimetric kit (Oxford Biomedical Research) that employs metallic cadmium for conversion of nitrate to nitrite prior to their quantification with Griess reagent. Due to the small size of the species, a low volume of blood (20-100 μl) was collected from the caudal vein of each anaesthetized male using a heparinised 2.5 ml syringe with a 27-gauge needle. Each sample was centrifuged (2000 rpm, 5 minute, 4°C) to collect the plasma, then stored at –80°C until used. For NO assay 10 μl of plasma were processed according to the manufacturer's protocol and the absorbance at 540 nm was determined with a microplate reader (AS1000 Packard SpectraCount). A standard curve was prepared with a stock nitrite standard. The assay was repeated twice for each blood sample, yielding a repeatability value of 0.81 ± 0.08 SE (Becker 1984), and the mean value was used for the analyses.

Previous studies helped us in determining the dosage appropriate to induced a rapid but transient immune stimulation (Haukenes & Barton 2004, Loyau et al. 2005, Lopez et al. 2009).

Mate-choice experiment

Design of male dummies

To design dummies head-crest colouration we used two extreme trait values (maximum and minimum) with the aim of increasing the females' chances of discerning differences. These values

corresponded to those recorded in the wild population (yellow patch area recorded on N=91 natural males, yellow colour intensity N= 42 natural males, Pizzolon 2010).

As described above the minimum value of yellow patch colour intensity was 1 (= R 255, G 200, B 000) and the maximum was 4 (= R 225, G 230, B 139). The minimum value of yellow patch size recorded in wild males was of 0.1 cm^2 and the maximum of 0.8 cm^2.

Dummies made from silicon fish-shaped lures obtained by injecting liquid silicon on a mould created on the body of a dead *S. pavo* male, and painted with non-toxic paints (Fig. S1 in Online Resource 2).

Mate-choice arena and experimental details

The experimental tank (60 x 35 x 34 cm) had a sandy bottom and was divided into two equal parts (60 x 17.5 x 34 cm) with a transparent Plexiglass partition: one section, provided with an artificial nest in the middle (a PVC tube, 15 cm long, 3 cm in diameter), hosted the female and the other section hosted two male dummies inside a PVC nest with heads protruding, so that females could clearly see the dummies' head crests (Fig. S2 in Online Resource 2).

Position of the two types of dummies was randomly swapped among replicates. A female was considered to respond to the dummies if she quitted the nest and performed, in front of the tank partition, the typical courtship displays of the species, i.e. rapid respiratory movements, pectoral fin fanning and display of the ventral region (Almada et al. 1995). Female preference was established on the basis of the time spent by the female in front of each dummy, within a choice zone of 17 x 5 cm near the tank partition (Fig. S2 in Online Resource 2). The time spent in front of the middle part of the partition, i.e. between the two dummies, or at the back of the aquarium was considered as no choice. If a female stayed in the nest, did not perform any courtship display within the first 15 min or spent less than 3 min in the choice zones the trial was discarded. Each female was used for a single trial that lasted for 30 minutes. In 32 out of 42 trials (first trial: 11 out of 15; second trial: 11 out of 13; third trial: 10 out of 14) females showed an interest in the dummies.

SUPPLEMENTARY RESULTS

Effect of immune treatment on head crest volume

To control if the slight modification in head crest size parameters (thickness, height, and area) recorded in LPS-treated males affect the whole structure expression, we computed its volume

trough an approximation of head crest to a conical figure with curving sides, described by a sine function, and with an elliptical base that progressively decreases in area but maintains the same proportion between major and minor axes. Head crest total volume does not change in response to the immune-challenge (paired-samples t-test: t= -1.52; p=0.14).

Effect of LPS dosage

Results proved the immunological effect of LPS dosage as similar levels of NO were detected before the treatment (LPS: 263.14 µM ± 52.96 SE; controls: 282.76 µM ± 48.81 SE) whereas NO concentration increased in LPS-injected males (415.34 µM ± 68.08) and decreased in controls (259.44 µM ± 41.89) after the injection (repeated measures ANOVA: time x group $F_{1,12}$= 5.58, P=0.036).

Table S1. Mean values of morphological and behavioural traits before and after the immune challenge in LPS and PBS (control) treated groups.

	**LPS-treated**			**CONTROLS**		
	**Pre-treatment** mean±st.dev.	**Post-treatment** mean±st.dev.	N	**Pre-treatment** mean±st.dev.	**Post-treatment** mean±st.dev.	N
**Morphological traits**						
Body weight (g)	12.28±6.37	11.87±5.84	21	10.820±4.17	10.29±4.01	21
Head crest area (cm^2)	0.553±0.397	0.536±0.402	21	0.484±0.298	0.480±0.303	21
Head crest colour area (cm^2)	0.306±0.242	0.201±0.268	21	0.277±0.161	0.241±0.136	21
Head crest colour intensity	3.095±0.889	1.333±0.856	21	2.857±0.793	2.476±1.030	21
Head crest height (cm)	0.503±0.205	0.474±0.217	21	0.484±0.161	0.476±0.168	21
Head crest thickness (cm)	0.116±0.033	0.130±0.040	21	0.129±0.026	0.109±0.028	21
Anal gland area (cm^2)	0.110±0.052	0.086±0.043	21	0.092±0.035	0.088±0.031	21
**Behavioural traits**						
Time outside nest (%)	17.78±20.90	17.09±20.75	21	17.41±23.66	22.53±31.31	21
N° attacks to female (n°/sec)	0.0034±0.0068	0.0132±0.0077	21	0.0073±0.0.0170	0.0077±0.0104	21
Reaction time to female (sec)	859±371	591±424	21	765±370	640±345	21

References

Almada VC, Gonçalves EJ, Oliveira R, Santos AJ (1995) Courting females: ecological constraints affect sex roles in a natural population of the blenniid fish *Salaria pavo*. Anim Behav 49: 1125-

1127.

Becker WA (1984) A manual of quantitative genetics. Washington DC: Pullman Academic Enterprises.

Färber A, Jahns P (1998) The xanthophyll cycle of higher plants: influence of antenna size and membrane organization. Biochim Biophys Acta 1363:47–58.

Haukenes AH, Barton BA (2004) Characterization of the cortisol response following an acute challenge with lipopolysaccharide in yellow perch and the influence of rearing density. J Fish Biol 64: 851-862.

López P, Gabirot M, Martín J (2009) Immune challenge affects sexual coloration of male Iberian wall lizards. J Exp Zool A Ecol Genet Physiol 311: 96–104.

Loyau A, Saint Jalme M, Cagniant C, Sorci G (2005) Multiple sexual advertisements honestly reflect health status in peacocks (*Pavo cristatus*). Behav Ecol Sociobiol 58:552-557.

Pizzolon M (2010) Multiple male traits and female choice in *Salaria pavo*. Ph.D. Thesis. Padova, Italy: University of Padova.

Sild E, Hõrak P (2009) Nitric oxide production: an easily measurable condition index for vertebrates. Behav Ecol Sociobiol 63: 959-966.
