# Supplementary material for: One trait, many signals: different information on male quality is enclosed within the same trait in a blenny fish
Source: Naturwissenschaften. 2012 Aug 17;99(10):863–7. doi: 10.1007/s00114-012-0959-4 (PMC3448905; doi:10.1007/s00114-012-0959-4)

SUPPLEMENTARY FIGURES

Figure S1

Photographs of a *S. pavo* natural male inside the nest (a) and of a male dummy designed for the mate-choice trials (b).

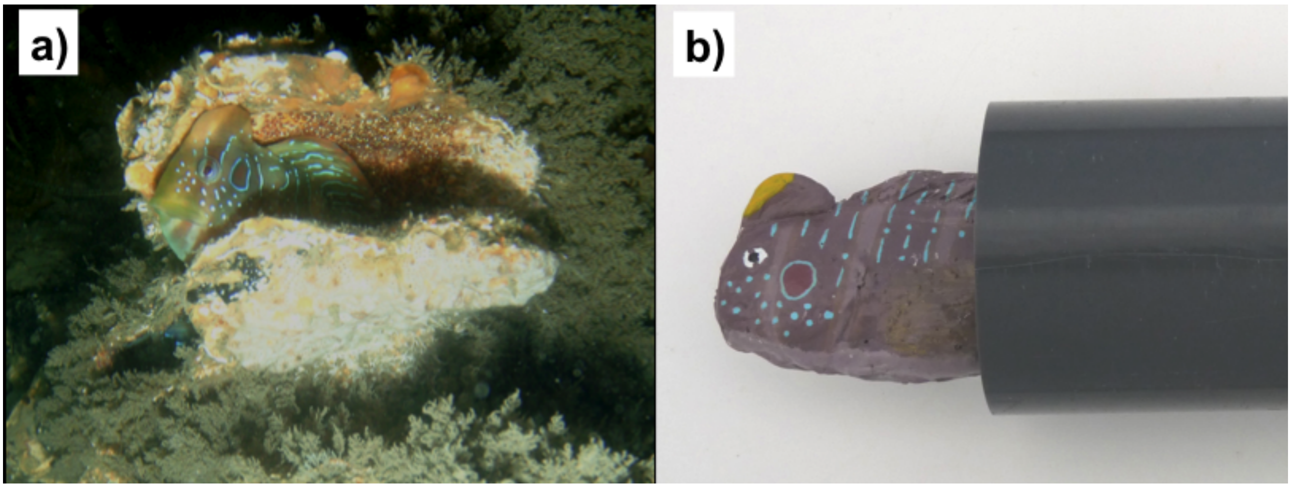

Figure S2

Schematic drawing of the mate-choice arena.

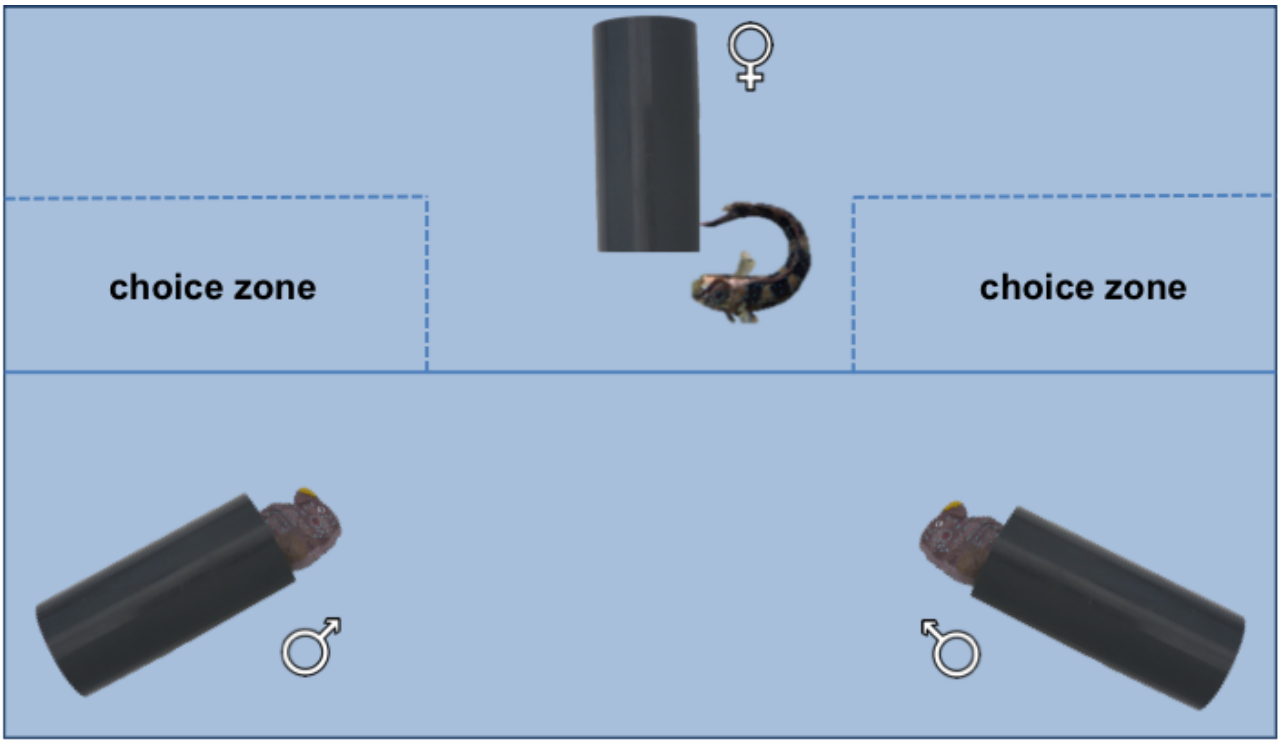

Supplement: Supplementary file 2 — (PDF 601 kb) [file 114_2012_959_MOESM2_ESM.pdf]
